# SheVari4^®^, a proprietary root extract from *Asparagus racemosus*, ameliorates menopausal health: insights from a randomized, double-blind, placebo controlled clinical trial

**DOI:** 10.29219/fnr.v70.14275

**Published:** 2026-07-21

**Authors:** Raktim Chattopadhyay, Subhankar Karmakar, Tandra R. Chakraborty, Manashi Bagchi, Samudra P. Banik, Debasis Bagchi

**Affiliations:** 1 Esperer Onco Nutrition Pvt Ltd, Thane, Navi Mumbai, Maharashtra, India; 2 Lakshya Hospital, Pune, Maharashtra, India; 3 Biology Department, College of Arts and Sciences, Adelphi University, Garden City, NY, USA; 4 Research & Development Department, Dr. Herbs LLC, Concord, CA, USA; 5 Department of Microbiology, Government General Degree College, Narayangarh, Rathipur, West Bengal, India; 6 Department of Pharmaceutical Sciences, Texas Southern University College of Pharmacy, Houston, TX, USA

**Keywords:** Menopausal Quality of Life, Asparagus racemosus, Shatavarin IV, SheVari4^®^, endocrine balance, psychosocial wellbeing

## Abstract

**Objective:**

Menopause marks the natural end of reproductive phase of women caused by decline in levels of estrogen, progesterone and other reproductive hormones. *Asparagus racemosus* (Shatavari) is a medicinal herb particularly known to be effective in alleviating women’s reproductive health. Shatavarin IV, a steroidal saponin and a primary bioactive component in this herb, acts as a phytoestrogen by modulating the ER-alpha/ER-beta signalling, the TrkB-BDNF axis, and the Hypothalamic-Pituitary-Gonadal (HPG) and Hypothalamic-Pituitary-Adrenal (HPA) axes. In this study, the efficacy of SheVari4^®^ on alleviating the menopause-specific endocrine dysfunction and subsequent impaired quality of life (QOL) was assessed through a randomized double-blind placebo-controlled parallel arm trial conducted on 60 pre-, peri-, and post-menopausal women.

**Methodology:**

Sixty women (age 50.0 ± 7.4 years) randomized in two equal groups were administered with 100 mg SheVari4^®^ or placebo capsules once daily for 8 weeks. Assessment of Menopause-Specific Quality of Life (MENQOL) along with domain scores across vasomotor, psychosocial, physical, and sexual function constituted primary endpoint analysis; changes in serum estradiol E2, Follicle Stimulating Hormone (FSH), progesterone, cortisol, Anti Mullerian Hormone (AMH), Sex Hormone-Binding Globulin (SHBG) and free testosterone were assessed as secondary endpoint analysis. Safety of the formulation was evaluated in terms of body mass index, blood biochemistry and liver function test.

**Results:**

SheVari4 induced a 42.8% reduction in MENQOL versus placebo. Significant improvements in functioning (vs. placebo) were observed, respectively, across vasomotor (53.1% vs. 14.5%), psychosocial (53.6% vs. 9.9%), physical (39.2% vs. 6.5%) and sexual (29.4% vs. 10.7%) domains. The formulation also increased E2 (+36.44% vs. –2.08%) and progesterone (+0.30% vs. –2.88%) coupled with marked reduction in levels of FSH (–38.18% vs. +12.97%) and cortisol (–26.87% vs. +17.16%). Concomitantly, AMH, SHBG, and free testosterone were also favorably modulated. Blood biochemistry, liver function tests, and vital signs confirmed the broad-spectrum safety of SheVari4^®^.

**Conclusion:**

SheVari4^®^ significantly improved menopause-specific QOL and endocrine parameters in peri- and post-menopausal women, with a well-established safety profile.

## Popular scientific summary

- Menopause leads to permanent inhibition of menstrual cycle and is responsible for a holistic decline in mental and physical health of women. In this study, we demonstrate the ameliorating effect of SheVari4^®^, a standardized root extract of Shatavari or *Asparagus racemosus* on perimenopausal and menopausal women. Daily consumption of 100 mg of the extract for 8 weeks led to significant improvement in menopausal health as assessed by MENQOL score and endocrine restoration. The formulation was safe to use and led to overall rejuvenation of health and metabolism.

Menopause represents the natural physiological confirmation of the end of reproductive phase in a woman’s life marked by the absence of periods for 12 consecutive months. The primary reason accounting for the onset of menopause is the significant decline in production of reproductive hormones estrogen and progesterone by ageing ovarian follicles. The anterior pituitary tries to compensate for this loss by producing more Follicle Stimulating Hormone (FSH) (1) and Luteinizing Hormone (LH) (2) leading to a perturbed feedback loop, endocrine imbalance and disruption of Hypothalamic-Pituitary-Ovarian (HPO) axis (3). Symptoms of menopause typically start appearing around 45 years, a condition referred to as perimenopause where menstrual cycles become irregular with frequent skipping of periods and continues within 1 year of menopause (4) with vasomotor symptoms including hot flashes, night sweats (5) and other Genitourinary syndromes (GSM) (6). Although classified as a natural physiological response of the body, menopause is often associated with a lot of health issues and ailments including cardiovascular diseases (7), sarcopenia (8), osteoporosis (9) as well as occasional cognitive dysfunctions leading to a gross decline in the body’s homeostasis. Menopause associated psychophysiological discomfort and decline in mental health has been the biggest challenge toward the well-being of women past their reproductive age (10). The psychological changes imparted by fall in estrogen levels severely jeopardize the daily chores in a woman’s life affecting sleep cycle, metabolic activity, concentration and work efficiency. This eventually leads to anxiety and a permanent state of depression (11) induced by fluctuations in the levels of serotonin and GABA (12). Besides the natural course of aging, menopause such as conditions can often be caused by specific medical interventions such as oophorectomy or surgical removal of ovaries (13), chemotherapy (14) and endometriosis (15).

Till date, the most popular curative strategy to treat menopausal distress has been hormone replacement therapy (HRT), or the collective set of procedures, which involve external administration of estrogen (along with progesterone if the patient has intact uterus) mostly in the form of oral, mucosal or epidermal absorption to make up for the deficit of production of these hormones in the body (16). HRT mimics the ovary in terms of production of estrogen and progesterone and works best for persons who are below 60 years of age or within 10 years of menopause. Beyond the age of 60, finite risk factors of venous thrombosis increase significantly (17). Possibilities of venous thrombosis also shoot up with individuals with greater body mass index (BMI) (18) as well as in persons with factor V Leiden inherited blood clotting disorder (both homozygous and heterozygous alleles) (19). Apart from this critical issue, HRT is also associated with a finite risk of cardiovascular ailments (20), dementia and breast cancer (21). Other minor but discomforting side effects of HRT include swelling with associated pain in breasts, sporadic vaginal bleeding, digestive problems with frequent bloating, headaches, mood swings, hair loss and occasional skin rashes.

In view of the concerning side-effect of HRT, alternative treatment options of menopausal symptoms involving the use of phytotherapeutic formulations had been in vogue since the last few decades (22). The most significant of these endeavors has been the exploitation of *Asparagus racemosus*, more popularly known as Shatavari, or the ‘Queen of the herbs’, a perennial shrub grown in abundance across the Indian sub-continent and Africa. The herb owes its name to its extraordinary potential in amelioration of a plethora of human ailments (23), particularly as an elixir of female reproductive health (24, 25). The primary bioactive constituent of Shatavari is Shatavarin IV (26), a steroidal saponin with structural resemblance to estrogens. *In silico* analyses had suggested that the molecule can successfully bind to estrogen β and γ receptors (27), which explains the strong phytoestrogenic potential of the molecule. Many independent studies had established that consumption of Shatavari root extract results in significant reduction of vasomotor symptoms associated with menopause (28, 29). Apart from its plausible role in the estrogenic pathway, recent studies in our laboratory had unearthed novel mechanistic insights to explain the therapeutic roles of Shatavari. A combination of docking and wet lab validation had demonstrated that Shatavarin IV can also augment the Tropomyosin Kinase B (TrkB)-BDNF signalling axis to dampen inflammatory response and associated oxidative stress (30). In the present studies, the potential of a standardized shatavari root extract, SheVari4^®^, in alleviating menopausal health was investigated through a double arm placebo-controlled study on a group of 60 women aged around 50 years. The study involved assessment of MENQOL, evaluation of hormonal levels and ascertaining safety parameters. It is worthwhile to mention here that the safety of this formulation was affirmed through an acute oral toxicity study in female Wistar rats, confirming LD50 > 2,000 mg/kg body weight (b.w.) and GHS Category 5 classification for SheVari4^®^ (31). Furthermore, Ames’ bacterial reverse mutation assay exhibited no cytotoxicity in either the histidine revertant colonies or the bacterial background lawn at a concentration of upto 5,000 µg/plate in any of the tester strains (unpublished data).

## Materials and methods

### Preparation of standardized Asparagus racemosus root extract (SheVari4^®^)

A standardized *A. racemosus* root extract (*SheVari4*^®^; light brown fine powder; >5% w/w shatavarin IV minimum specification (HPLC); CR/SHA/2025/NOV/014; Mfg date: Nov 2025; Exp date: Oct 2028) was prepared utilizing a unique water–ethanol extraction technology in a GMP-NSF certified facility at Chemical Resources, Panchkula, Haryana, India. SheVari4^®^ was stored at 15–25°C in a cool and dry place, protected from light and moisture.

### Estimation of SheVari4^®^ by HPLC

SheVari4^®^ (54.89 mg) was dissolved in 25 mL methanol, vortexed for 30 s and sonicated for 30 min at room temperature, mixed well, filtered through a 0.45 μm syringe filter, and injected (injection volume 0.2 mL) into an Liquid Chromatography–Mass Spectrometry (LCMS) equipped with an Evaporative Light Scattering Detector (ELSD) detector and Luna C18 (2) 5 mm (150 × 2.0 mm) column at a flow rate of 0.4 mL/min and temperature of 60°C using a mobile phase of (a) trifluoroacetic acid: water (0.1: 99.9; v/v); (b) acetonitrile. The retention time of Shatavarin IV was 18.3749 min and the percentage of Shatavarin IV content in SheVari4^®^ was determined employing the formula (test area × concentration of standard × % potency of standard)/standard area × concentration of test.

#### Residual solvent, pesticides and heavy metals

SheVari4^®^ meets the limits set forth for residual solvents as per class I, class II, and class III solvents in United States Pharmacopoeia (USP) <467>; and residual solvents option 2; thus, SheVari4^®^ was found to be free from residual solvents employed during the production process. SheVari4^®^ also conforms to the limits set forth for pesticide residue analysis as per USP <561>; and no mercury, arsenic or cadmium was detected in the sample, and lead was detected 0.0033 ppm and total heavy metal content was reported to be 0.0033 ppm as determined by Inductively Coupled Plasma–Mass Spectrometry (ICPMS). Thus, SheVari4^®^ was also found to be free from pesticides and heavy metals.

#### Placebo and Shevari4^®^ capsules

SheVari4^®^ was filled in 100 mg hard gelatin capsules (Size 0, opaque white; Batch # CR/SHA/2025/NOV/014; Mfg date: Nov 2025; Exp date: Oct 2028). In conjunction, matching placebo capsules containing microcrystalline cellulose at 100 mg (Size 0, opaque white; Batch No. CR/SHA/2025/NOV/015) were manufactured in the same facility and stored at 15–25°C in a cool and dry place, protected from light and moisture. Placebo capsules were indistinguishable from the SheVari4^®^ capsules in weight, appearance, size, color, weight, and packaging.

### Ethical and Institutional Ethics Committee approvals

This prospective, randomized, double-blind, placebo-controlled, parallel-group pilot clinical investigation (Protocol No. CT-CE-CEPHAM-04-1225) was conducted in accordance with the guidelines for Good Clinical Practices (GCP)(E6 R2), Declaration of Helsinki (2013 revision) and International Council for Harmonization (ICH) guidelines for Good Clinical Practice guidelines. The study was sponsored by Cepham Inc. (Somerset, NJ, USA) and conducted in Lakshya Hospital (Pune, Maharashtra, India). Subject confidentiality was strictly maintained. All subjects were recruited as per the inclusion and exclusion criteria approved by the Institutional Ethics Committee (IEC) of Lakshya Hospital (Pune, Maharashtra, India) and subject confidentiality was strictly maintained (IEC approval# EC/NEW/LPUNE-567/2026).

The study protocol, informed consent form, and all related documents were submitted to and approved by the IEC before any study procedures began. Written and audio-video informed consent was obtained from all subjects, who were given adequate time to consider enrollment and were told they could withdraw at any time without penalty. All subject data were handled in line with applicable data protection regulations, with subjects identified only by a unique number. The sponsor provided insurance coverage for study-related injuries, and subjects received reasonable compensation for time and travel in accordance with local ethics committee approval. Study data were collected using electronic case report forms within a validated data capture system.

### Recruitment of subjects

**Fig. 1:**
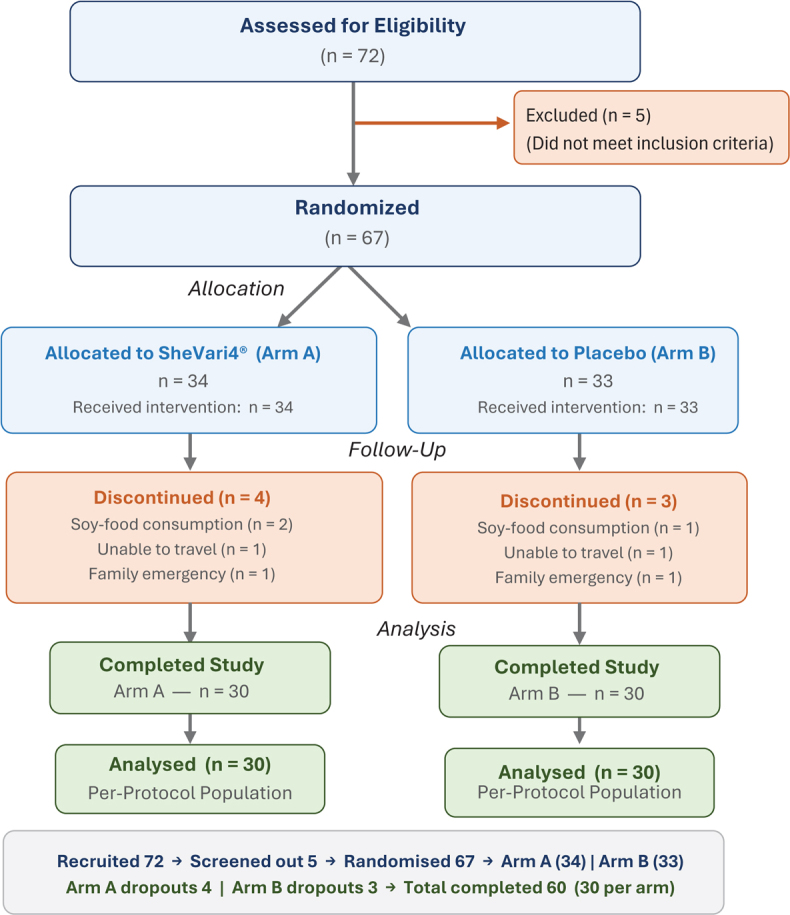
CONSORT Diagram showing allocation of women showing menopausal symptoms randomized as per PROC plan block randomization into Arm A (SheVari4^®^, *n* = 34) and Arm B (Placebo, *n* = 33).

A total number of 72 women were initially selected for this pilot study to assess the efficacy of SheVari4^®^ out of which 5 were excluded while screening as shown in the CONSORT diagram (Fig. 1). The remaining 67 patients were randomized as per PROC plan block randomization into Arm A (SheVari4^®^, *n* = 34) and Arm B (Placebo, *n* = 33) using a computer-generated schedule with permuted blocks of 4 and 6, prepared by an independent statistician and maintained by an unblinded pharmacist. A further four individuals from Arm A and 3 from Arm B dropped out from the study resulting into a final number of 30 patients in each arm. Allocation concealment was ensured with sequentially numbered, opaque, sealed envelopes. The study was fully double-blind. The blinding code was held by the sponsor’s quality assurance department and broken only at study completion for data analysis, or in a medical emergency via a 24-h hotline. Both participants and all outcome assessors remained blinded throughout, and any unblinding events were documented and reported.

### Concomitant medication

All study participants regularly and routinely maintained the records of intake of all concomitant prescription medications, over-the-counter (OTC) medications and non-prescription medications during the investigation and all these were recorded on the case report forms.

In Menopause-Specific Quality of Life (MENQOL), prohibited medications included HRT, SERMs, hormonal contraceptives, phytoestrogen supplements, black cohosh, dong quai, evening primrose oil, and any botanical marketed for menopausal symptoms. However, stable doses of antihypertensives, lipid-lowering agents, thyroid medications, and OTC analgesics were permitted, provided the dose had been stable for at least 4 weeks before screening. All concomitant medications were recorded at each visit.

### Study duration

The treatment was conducted over a period of 8 consecutive weeks, followed by a 2-week follow-up, for a total of 10 weeks. Visits were scheduled at screening (Day −14 to −1), baseline (Day 1, randomization), weeks 2, 4, 6, and 8 (end of treatment), and a final follow-up at week 10 (Day 71 ± 3). Subjects could also exit at an early termination visit at any time.

### Participants’ consent

Study participants were recruited as per the IEC-approved inclusion and exclusion criteria. All recruited participants reviewed, understood, discussed, and duly signed the IEC-approved health questionnaire and consent forms. Adverse events monitoring was enforced.

### Study compliance

Both SheVari4^®^ and placebo capsules were stored at room temperature in a dark, cool, and dry place, and kept away from direct sunlight and moisture. Principal investigator (PI) and the project coordinators distributed the SheVari4^®^ and placebo capsules to the recruited subjects, while individual data entry was endorsed by the PI. Thus, PI regularly signed off the investigational product (IP) accountability log. All recruited participants were given a daily diary for recording the details of capsule and food intake, regular physical activities, and any observed untoward or adverse events, and get their diaries endorsed by the study coordinators and PI. Each subject received 65 capsules (5 extra capsules were provided) in a sealed bottle. Details of the capsule distribution was recorded in the IP accountability log, and endorsed by the PI. IP accountability log was made available by the PI during the technical audit.

### Study confidentiality

To optimize selection bias in this prospective, single-center, randomized, double-blind, placebo-controlled, parallel-group clinical trial, allocation concealment was conducted on the enrolled study subjects, investigators, and study coordinators responsible for the assessment of the enrolled subjects entering the trial.

### Dosage regimen

Each capsule contained 100 mg of either SheVari4^®^ or placebo and the capsules were advised to be taken once daily with the evening meal, orally with one cup of water, over a period of 8 consecutive weeks. Each subject received 65 capsules per kit (including five additional capsules as buffer) were classified as protocol deviators.

### Study discontinuation clause

In the events of untoward or adverse events (as exhibited in the safety assessment clause), the regulation for ‘stopping’ of ‘clinical investigation’ or ‘discontinuation clause’ were applicable.

### Inclusion and exclusion criteria

Women (39–65 years) experiencing menopausal symptoms were enrolled. The study included premenopausal, perimenopausal, and postmenopausal women. An additional 10% of subjects were screened to account for screen failures.

### Primary end point: the MENQOL

In 1996, a self-reported validated tool termed as MENQOL was developed (32), which consists of 29-item questionnaire scored on a 1–8 Likert scale, to evaluate the impact of menopausal symptoms on the quality of life (QOL). In MENQOL, higher scores reflect greater symptom burden and lower scores indicate improvement.

The vasomotor domain of MENQOL consists of three items: hot flushes, night sweats, and sweating; while the psychosocial domain covers seven items, including personal dissatisfaction, anxiety, poor memory, reduced accomplishment, depression, impatience, and social withdrawal. The physical domain spans 16 items covering flatulence, muscle and joint aches, fatigue, sleep difficulties, headaches, declining strength and stamina, energy loss, skin dryness, weight gain, facial hair, skin texture changes, bloating, low backache, urinary frequency, and urinary incontinence; and finally, the sexual domain includes three items: change in sexual desire, vaginal dryness during intercourse, and avoidance of intimacy.

Secondary endpoints included MENQOL subscale scores at Week 8 and the percentage change in fasting serum E2, FSH, LH, progesterone, cortisol, prolactin, AMH, Sex Hormone-Binding Globulin (SHBG), and free testosterone. A blood biochemistry safety panel was also collected, covering liver function tests (Alanine Aminotransferase [ALT], Aspartate Aminotransferase [AST], bilirubin), kidney function tests (serum creatinine, eGFR, blood urea nitrogen [BUN]), complete blood count, comprehensive metabolic panel, and a 12-lead ECG, along with vital signs and body weight.

The exploratory analyses evaluated the correlations between MENQOL symptom improvement and hormone changes, identified potential responder subgroups by baseline menopausal status, and assessed whether treatment effects persisted through the week 10 follow-up visit.

All blood samples for hormonal level analyses were collected between 08:00 and 09:00 AM after a minimum 12-h overnight fast (water was permitted). Before collection, subjects rested and seated for 15 min, avoided vigorous exercise for at least 30 min, and waited at least 1 h after waking to avoid confounding by the cortisol awakening response. Samples were not drawn during acute illness or periods of unusual stress. Blood was collected in serum separator tubes (SST) (15 mL total) and allowed to clot for 30 min at room temperature. Serum was centrifuged at 2,000–3,000 × rpm for 10 min, aliquoted into labeled cryovials with at least 2 aliquots per hormone panel, stored at −80°C, and shipped to the central laboratory on dry ice.

For perimenopausal subjects, still on cycling regime, blood was drawn on days 2–5 of the menstrual cycle during the early follicular phase, while for those not on cycling regime, blood was collected at a consistent time on any day across all visits. Protocol for blood collection for postmenopausal women was similar. Table 2 demonstrates the hormone/biomarkers, their reference ranges and medical significance.

**Table 1:** Inclusion and exclusion criteria parameters

*Inclusion criteria*
Female, aged 39–65 years BMI 18.5–29.9 kg/m^2^ Resting SBP < 140 mmHg and DBP < 90 mmHg, with resting heart rate < 90 bpm Menopausal symptoms for ≥ 3 months Menopause Rating Scale total score ≥ 10 at screening and baseline ≥ 2 Hot flushes per day or ≥ 14 per week during screening Premenopausal with documented menopausal symptoms, or perimenopausal (irregular menstrual cycles for ≥12 months), or postmenopausal (amenorrhea for ≥ 12 consecutive months) Willing to maintain existing dietary and physical activity patterns throughout Written and audio-video informed consent obtained
*Exclusion criteria*
Use of HRT, SERMs, or hormonal contraceptives within 3 months before screening Use of phytoestrogen supplements, black cohosh, dong quai, or evening primrose oil within 4 weeks History of hormone-sensitive malignancy or estrogen-dependent neoplasia Undiagnosed abnormal vaginal bleeding History of thromboembolic disorders, stroke, or myocardial infarction Uncontrolled hypertension (SBP > 160 mmHg or DBP > 100 mmHg) Uncontrolled diabetes (HbA1c > 9%) Active liver disease or ALT/AST > 2× ULN Severe renal impairment (eGFR < 30 mL/min/1.73 m^2^) History of hysterectomy or pelvic radiotherapy Chronic inflammatory conditions such as rheumatoid arthritis, Crohn’s disease, lupus, or HIV/AIDS Dietary consumption of soya-containing foods Alcohol intake above 2 standard drinks per day Current or recent history of smoking Participation in another clinical trial within 30 days Known hypersensitivity to *Asparagus racemosus* or related plants

**Table 2:** Hormone/biomarker panel: reference ranges, and medical significance

Biomarkers/ hormones	Unit	Permissible range	Medical significance
Estradiol (E_2_)	pg/mL	Premenopausal: 30–400; Postmenopausal: < 30	Primary ovarian estrogen; phytoestrogenic activity biomarker
Luteinizing hormone (LH)	mIU/mL	Premenopausal: 2–15; Postmenopausal: 15–60	Gonadotropin status
Follicle-stimulating hormone (FSH)	mIU/mL	Premenopausal: 3–10; Postmenopausal: 30–120	HPG axis feedback and ovarian reserve biomarker
Progesterone	ng/mL	Follicular: < 1; Luteal: 5–20	Ovulation confirmation; luteotrophic marker
Free Testosterone	pg/mL	0.2–5.0	Androgen status and sexual function biomarker
Sex Hormone-Binding Globulin (SHBG)	nmol/L	18–144	Hormone binding capacity; hepatic estrogenic effect
Anti-Müllerian Hormone (AMH)	ng/mL	Premenopausal: 1–3.5; Postmenopausal: < 0.1	Ovarian reserve and granulosa cell function
Cortisol (AM)	µg/dL	6–23	HPA axis stress and adaptogenic marker
Prolactin	ng/mL	2–29	Pituitary function marker

The distinction between cycling and non-cycling perimenopausal subjects is clinically sound and the Day 2–5 follicular-phase window is the correct standard for hormonal biomarker collection (FSH, E2, Progesterone).

### Safety and toxicity assessment

Safety assessment was assessed at each visit through adverse event reporting coded by MedDRA (Medical Dictionary for Regulatory Activities) system organ class and preferred term. Laboratory testing covered liver function tests (ALT, AST, ALT/AST ratio, and bilirubin), kidney function tests (serum creatinine, estimated Glomerular Filtration Rate [eGFR], and Blood Urea Nitrogen [BUN]), a complete blood count, a comprehensive metabolic panel, and a 12-lead Electrocardiogram (ECG). Vital signs and body weight were also recorded.

### Adverse events

All study participants were instructed to record any or all types of adverse events in their daily diaries. All subjects were critically questioned if they had experienced any untoward problems or difficulties during their routine visits to the clinical study center. Overall, adverse event monitoring was strictly enforced.

### Statistical analyses

Analyses were conducted utilizing Python 3.x with SciPy and Pandas, against a pre-specified statistical analysis plan. The primary model was a linear mixed-effects model for repeated measures incorporating fixed effects of treatment, visit, and their interaction, with baseline score as a covariate and an unstructured covariance matrix. Both intent-to-treat and per-protocol populations were analyzed, and results here reflect the intent-to-treat population. PROC PLAN was used for generating randomization schedules and treatment assignment plans (33). Within-group changes were assessed with paired two-tailed student’s *t*-tests, and between-group comparisons used independent-samples two-tailed *t*-tests. Effect sizes are reported as Cohen’s d, where values of 0.2, 0.5, and 0.8 represent small, medium, and large effects. All primary change scores include 95% confidence intervals (CIs). Statistical significance was set at *P* < 0.05, two-tailed. The per-protocol population comprised 60 subjects (30 per arm) who completed the 8-week intervention. No imputation was applied for the per-protocol analysis. A full intent-to-treat analysis (*n* = 67) applying last observation carried forward will be reported in the subsequent studies.

## Results

**Fig. 2:**
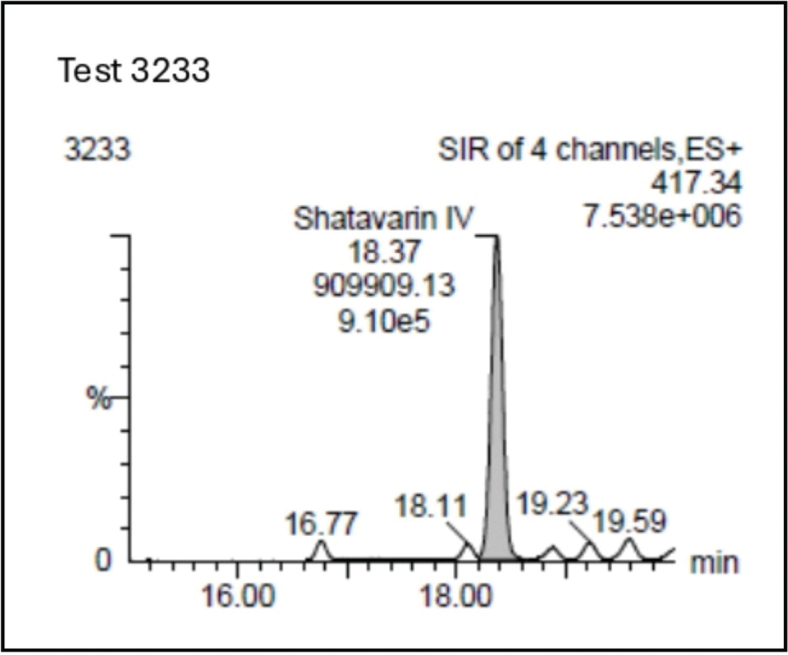
HPLC chromatogram of ethanol:water (45:55 v/v) extract SheVari4^®^ (run as per conditions elaborated in Materials and Methods section).

This study assessed the safety and efficacy of SheVari4^®^, a novel, proprietary extract from *A. racemosus* (Shatavari), in alleviating women’s MENQOL and a panel of hormonal biomarkers in 60 peri- and post-menopausal women. SheVari4^®^was found to contain 7.094% Shatavarin IV, as revealed by HPLC (Fig. 2). Baseline demographic and medical data were almost identical between the SheVari4^®^ and placebo groups (Table 3).

**Table 3:** Baseline demographic, anthropometric, and clinical characteristics of study participants

Parameters	SheVari4^®^ (Arm A)(*n* = 30) Mean ± SD	Placebo (Arm B)(*n* = 30) Mean ± SD	*P* (two-tailed)	Statistical test
Age (years)	52.37 ± 6.39	50.37 ± 4.21	0.158	*t*-test
Weight, pre-treatment (Kg)	62.55 ± 7.41	61.20 ± 6.06	0.444	*t*-test
Weight, post-treatment (Kg)	62.33 ± 7.41	61.32 ± 5.53	0.549	*t*-test
BMI, pre-treatment (Kg/m^2^)	24.71 ± 2.50	24.39 ± 1.75	0.573	*t*-test
BMI, post-treatment (Kg/m^2^)	24.84 ± 2.59	24.39 ± 1.59	0.421	*t*-test
BMI % change	+0.93 ± 9.69	+0.30 ± 7.24	0.774	*t*-test
Height (m)	1.584 ± 0.063	1.585 ± 0.052	0.955	*t*-test
Systolic BP (mmHg)	122.6 ± 3.6	121.2 ± 3.4	0.136	*t*-test
Diastolic BP (mmHg)	81.7 ± 2.1	81.7 ± 2.0	0.950	*t*-test
Pulse rate (bpm)	69.6 ± 4.1	70.4 ± 4.1	0.455	*t*-test

Values are expressed as mean ± SD. BMI within-arm change: Arm A *P* = 0.754, Arm B *P* = 0.999 (paired *t*-test). Menopausal status from clinical records (*χ*^2^ = 1.600, df = 2, *P* = 0.449). No baseline parameter differed significantly between arms, confirming balanced randomization.

### MENQOL data

Sixty women (age 50.0 ± 7.4 years) were randomized in two equal groups and administered with 100 mg SheVari4^®^ or placebo capsules/day over a period of 8 weeks. SheVari4^®^ treatment produced statistically significant changes improvements in all four MENQOL domains as compared to the placebo, with all between-arm comparisons reaching *P* < 0.0001 (Table 4). SheVari4^®^ group were highly significant (*P* < 0.0001 for all domains).

**Table 4:** MENQOL domain scores: baseline, post-treatment, and percentage change

Domain	SheVari4^®^ Group				Placebo Group				Cohen’s
	Baseline	Post-treatment	Change (95% CI)	Group (% ↓)	Baseline	Post-treatment	Change Δ (95% CI)	Group (% ↓)	
Total MENQOL	1.971 ± 0.442	1.128 ± 0.390	0.844 (0.763, 0.924)	42.8%	1.714 ± 0.610	1.615 ± 0.567	0.099 (0.017, 0.180)	5.8%	3.43***
Vasomotor	2.678 ± 0.675	1.256 ± 0.461	1.422 (1.246, 1.598)	53.1%	2.844 ± 1.046	2.433 ± 0.780	0.411 (0.213, 0.609)	14.5%	2.02***
Psychosocial	1.876 ± 1.118	0.871 ± 0.616	1.005 (0.739, 1.271)	53.6%	1.486 ± 1.050	1.338 ± 0.872	0.148 (0.011, 0.284)	9.9%	1.52***
Physical	1.654 ± 0.601	1.006 ± 0.474	0.648 (0.543, 0.752)	39.2%	1.415 ± 0.548	1.323 ± 0.566	0.092 (0.019, 0.164)	6.5%	2.31***
Sexual	3.178 ± 1.163	2.244 ± 0.973	0.933 (0.706, 1.161)	29.4%	2.711 ± 1.035	3.000 ± 1.058	−0.289 (−0.461, −0.117)	−10.7%	2.26***

Data are expressed as mean ± SD.

*** *P* < 0.0001 (between-arm comparison).

Δ = reduction in score (positive value = improvement). Negative % in the placebo group in sexual domain indicates worsening. All within-group changes in the SheVari4^®^ group: *P* < 0.0001.

The total MENQOL score reduced by 42.8% in SheVari4^®^ group (1.971 ± 0.442 to 1.128 ± 0.390; Δ = 0.844, 95% CI: 0.763–0.924; *P* < 0.0001), as compared with only 5.8% in the placebo (*P* = 0.0192). The between-group difference was highly significant (*P* < 0.0001; Cohen’s *d* = 3.43), reflecting a remarkable treatment effect (Table 4).

SheVari4^®^ reduced the vasomotor domain score by 53.1% (2.678 ± 0.675 to 1.256 ± 0.461; *P* < 0.0001; Cohen’s *d* = 2.02), as compared to 14.5% in placebo. Within this domain, hot flushes reduced from a mean of 3.20 to 1.43 on the 8-point scale, night sweats from 2.93 to 1.10, and sweating from 1.90 to 1.23 (all *P* < 0.003) (Table 4).

The psychosocial domain improved by 53.6% in the SheVari4^®^ group (1.876 ± 1.118 to 0.871 ± 0.616; *P* < 0.0001; Cohen’s *d* = 1.52), as compared to 9.9% in the placebo group, reflecting meaningful relief from anxiety, depression, poor memory, and interpersonal difficulties.

The physical domain score reduced by 39.2% in the SheVari4^®^ group (1.654 ± 0.601 to 1.006 ± 0.474; *P* < 0.0001; Cohen’s *d* = 2.31), as compared to 6.5% in the placebo group, pointing to reductions in fatigue, joint pain, and genitourinary symptoms (Table 4).

The most significant contrast between arms appeared in the sexual domain, where SheVari4^®^ induced a 29.4% improvement (3.178 ± 1.163 to 2.244 ± 0.973; *P* < 0.0001; Cohen’s *d* = 2.26), while the placebo group showed a significant decline of 10.7% (2.711 ± 1.035 to 3.000 ± 1.058; *P* = 0.0018), widening the treatment contrast considerably (Table 4).

### Findings on hormonal biomarkers

SheVari4^®^-supplemented changes in fasting serum E2, FSH, progesterone, cortisol, AMH, SHBG, and free testosterone were assessed in 60 women (placebo = 30; treatment group [SheVari4^®^] = 30) at the beginning and completion of 8 weeks of treatment. Administration of SheVari4^®^-induced modest to significant changes in the levels of all hormonal biomarkers except AMH and progesterones compared to placebo (Table 5).

**Table 5:** Effects on hormonal biomarkers: baseline, week 8 post-treatment, and percentage change

Biomarkers	SheVari4^®^ Group (Arm A, *n* = 30)			Placebo Group (Arm B, *n* = 30)			Within-Arm p (paired *t*-test, A; B)	Between-Arm p (ind. *t*-test on %Δ)
	Baseline Mean ± SD	Week 8 Mean ± SD	% Change Mean ± SD	Baseline Mean ± SD	Week 8 Mean ± SD	% Change Mean ± SD		
Estradiol (E_2_) (pg/mL)	28.92 ± 8.78	37.17 ± 9.37	+36.44 ± 41.51%	28.26 ± 10.74	25.84 ± 10.51	−2.08 ± 39.22%	< 0.0001; 0.228	0.0005
FSH (mIU/mL)	44.37 ± 15.82	26.02 ± 13.89	−38.18 ± 40.41%	39.27 ± 14.65	39.37 ± 12.02	+12.97 ± 56.07%	< 0.0001; 0.967	0.0001
Progesterone (ng/mL)	0.617 ± 0.251	0.617 ± 0.249	+0.30 ± 7.63%	0.676 ± 0.275	0.611 ± 0.251	−2.88 ± 40.33%	0.931; 0.145	0.672
Cortisol (µg/dL)	12.25 ± 3.21	8.69 ± 2.88	−26.87 ± 24.64%	12.63 ± 3.48	13.54 ± 2.33	+17.16 ± 52.15%	< 0.0001; 0.167	0.0001
AMH (ng/mL)	1.015 ± 0.516	1.022 ± 0.525	+0.64 ± 6.70%	1.005 ± 0.439	1.179 ± 0.579	+34.24 ± 74.51%	0.622; 0.067	0.017
SHBG (nmol/L)	55.41 ± 17.76	71.88 ± 17.96	+46.70 ± 82.14%	58.76 ± 17.06	55.03 ± 16.88	−3.80 ± 26.87%	< 0.0001; 0.150	0.002
Free Testosterone (pg/mL)	1.970 ± 0.971	1.156 ± 0.707	−36.31 ± 42.14%	2.204 ± 0.760	2.315 ± 0.713	+15.45 ± 53.03%	< 0.0001; 0.475	0.0001

Values are expressed as mean ± SD. % Change computed per-subject then averaged. Within-arm p: paired two-tailed *t*-test for each arm separately.

Between-arm p: independent samples *t*-test on percentage change values.

SheVari4^®^ produced a mean increase in serum estradiol (E2) of +36.44 ± 41.51% in the treatment arm (28.92 ± 8.78 pg/mL at baseline to 37.17 ± 9.37 pg/mL at Week 8; within-group *P* < 0.0001), compared with a non-significant decline of 2.08 ± 39.22% in the placebo arm (28.26 ± 10.74 to 25.84 ± 10.51 pg/mL; within-group *P* = 0.227; between-arm *P* = 0.0005). The substantial inter-subject variability reflected in the standard deviations is consistent with the mixed menopausal-stage composition of the study population, in which premenopausal, perimenopausal, and postmenopausal subjects contribute markedly different hormonal baselines; the directional signal and between-arm significance are nonetheless robust. This E2 restoration is consistent with phytoestrogenic modulation of the HPG axis by Shatavarin IV acting on estrogen receptor subtypes.

Concordant with the E2 elevation, FSH declined by 38.18 ± 40.41% in the SheVari4^®^ group (44.37 ± 15.82 to 26.02 ± 13.89 mIU/mL; within-group *P* < 0.0001), whereas FSH remained essentially unchanged in the placebo arm, recording a non-significant change of +12.97 ± 56.07% (39.27 ± 14.65 to 39.37 ± 12.02 mIU/mL; within-group *P* = 0.967; between-arm *P* = 0.0002). The parallel rise in E2 and fall in FSH constitute the expected hormonal signature of restored negative HPG feedback, wherein elevated circulating estrogen suppresses hypothalamic GnRH secretion and anterior pituitary FSH release, and together represent the most compelling endocrine evidence for phytoestrogenic activity in this study.

The most clinically notable finding across all biomarkers was a 26.87 ± 24.64% reduction in morning serum cortisol in the SheVari4^®^ group (12.25 ± 3.21 to 8.69 ± 2.88 µg/dL; within-group *P* < 0.0001), against an increase of 17.16 ± 52.15% in the placebo arm (12.63 ± 3.48 to 13.54 ± 2.33 µg/dL; within-group *P* = 0.167; between-arm *P* = 0.0001). This represents the largest absolute effect magnitude among all measured biomarkers, and is consistent with HPA axis adaptogenic activity attributable to Shatavarin IV. The divergent cortisol trajectories, with decline in the treatment arm and corresponding upward drift in the placebo arm, also support the broader interpretation that untreated menopausal transition is associated with progressive HPA dysregulation, and SheVari4^®^ actively attenuates this process.

Serum progesterone showed minimal change in both arms over the 8-week period, with the SheVari4^®^ group recording a mean change of +0.30 ± 7.63% (0.617 ± 0.251 to 0.617 ± 0.249 ng/mL; within-group *P* = 0.931) and the placebo group recording a change of −2.88 ± 40.33% (0.676 ± 0.275 to 0.611 ± 0.251 ng/mL; within-group *P* = 0.145). The between-arm difference did not reach statistical significance (*P* = 0.672), indicating that a single daily dose of 100 mg SheVari4^®^ over 8 weeks does not produce a measurable luteotropic effect on progesterone secretion in this population, a finding that merits further investigation in longer-duration or higher-dose trials.

AMH increased marginally and insignificantly in the SheVari4^®^ arm (+0.64 ± 6.70%; 1.015 ± 0.516 to 1.022 ± 0.525 ng/mL; within-group *P* = 0.621) while the placebo arm exhibited a numerically larger, though non-significant within-group increase (+34.24 ± 74.51%; 1.005 ± 0.439 to 1.179 ± 0.579 ng/mL; within-group *P* = 0.067). The between-arm comparison was statistically significant (*P* = 0.017), though this result should be interpreted cautiously given the markedly elevated standard deviation in the placebo arm, which likely reflects extreme inter-individual variability in residual ovarian reserve across the heterogeneous study population. Non-parametric sensitivity analysis (Mann-Whitney U) yielded *P* = 0.067 for this comparison, suggesting the parametric result may overstate the precision of the finding.

SHBG increased significantly in the SheVari4^®^ group (+46.70 ± 82.14%; 55.41 ± 17.76 to 71.88 ± 17.96 nmol/L; within-group *P* < 0.0001), while declining modestly and non-significantly in the placebo arm (−3.80 ± 26.87%; 58.76 ± 17.06 to 55.03 ± 16.88 nmol/L; within-group *P* = 0.150; between-arm *P* = 0.002). The large standard deviation in the SheVari4^®^ SHBG change reflects the well-established high inter-individual variability of SHBG responses to estrogenic stimulation, particularly across mixed menopausal stages, and does not attenuate the clinical significance of the directional effect. Elevated SHBG is consistent with the hepatic estrogenic response to Shatavarin IV and serves as an additional mechanistic marker of phytoestrogen activity.

In alignment with the SHBG elevation, free testosterone declined by 36.31 ± 42.14% in the SheVari4^®^ group (1.970 ± 0.971 to 1.156 ± 0.707 pg/mL; within-group *P* < 0.0001), while increasing non-significantly by +15.45 ± 53.03% in the placebo arm (2.204 ± 0.760 to 2.315 ± 0.713 pg/mL; within-group *P* = 0.475; between-arm *P* = 0.0001). This reduction in free testosterone is physiologically coherent: rising SHBG increases the sex-hormone binding capacity of plasma, thereby reducing the unbound androgen fraction. Far from being an adverse effect, reduced free testosterone in the menopausal context is associated with improved vasomotor symptom profiles, and the mechanistic coupling of the SHBG increase and free testosterone decline provides further internal consistency to the phytoestrogenic action of SheVari4^®^.

### Clinical biochemistry

No statistically significant changes in BMI, blood pressure, or pulse rate were observed in either arm (Table 1). Findings confirm that SheVari4^®^ is weight-neutral and does not adversely affect cardiovascular parameters. The preclinical safety profile, with LD50 > 2,000 mg/kg and GHS Category 5 classification, further supports the clinical tolerability observed.^26^

Liver, kidney and cardiovascular function tests (Serum glutamic-oxaloacetic transaminase [SGOT], serum glutamic-pyruvic transaminase [SGPT], alkaline phosphatase [ALP], BUN, and creatine kinase [CK]) were conducted at the beginning and completion of 8-week of supplementation in both placebo- and SheVari4^®^ groups (Table 6). A slight decreasing trend was observed in the SheVari4^®^ group at the completion of 8-weeks of treatment as compared to the placebo group (Table 6). SheVari4^®^ provided a lowering effect of CK level following supplementation over a period of 8-weeks. During adulthood, CK levels generally reduce in women, but may rise after menopause. Reduction of estrogen level after menopause leads to an increase in CK level (34). Thus, SheVari4^®^ supplementation may provide the similar membrane stabilizing effect that natural estrogen used to provide.

**Table 6:** Effect of placebo and SheVari4^®^ on liver, kidney, and muscle function indices: baseline, week 8 post-treatment, and percentage change

Biomarkers	Normal range	SheVari4^®^ group (Arm A, *n* = 30)			Placebo group (Arm B, *n* = 30)			Within-Arm p (A; B)	Between-Arm p (Week 8)
		Baseline Mean ± SD	Week 8 Mean ± SD	% Change Mean ± SD	Baseline Mean ± SD	Week 8 Mean ± SD	% Change Mean ± SD		
SGOT / AST (U/L)	10–40	27.50 ± 5.15	25.47 ± 4.48	−7.10 ± 4.06%	27.97 ± 5.44	28.53 ± 5.49	+2.17 ± 3.85%	< 0.0001; 0.006	0.021
SGPT / ALT (U/L)	7–56	24.23 ± 5.32	22.33 ± 5.25	−8.03 ± 6.03%	25.50 ± 4.56	26.33 ± 4.47	+3.56 ± 4.95%	< 0.0001; 0.0009	0.002
ALP (U/L)	44–147	72.77 ± 13.18	71.10 ± 12.63	−2.12 ± 3.83%	71.20 ± 10.58	72.50 ± 10.71	+1.92 ± 3.65%	0.001; 0.006	0.645
BUN (mg/dL)	7–20	10.93 ± 2.00	9.90 ± 1.32	−7.84 ± 13.89%	11.73 ± 1.87	11.90 ± 1.37	+2.66 ± 10.84%	0.001; 0.455	< 0.0001
CK (U/L)	22–198	78.43 ± 17.03	74.70 ± 15.84	−4.62 ± 2.03%	78.27 ± 14.16	78.13 ± 13.01	+0.16 ± 3.19%	< 0.0001; 0.766	0.363

Values are expressed as mean ± SD; Within-arm p: paired two-tailed *t*-test for each arm separately (Arm A value; Arm B value).

Between-arm p: independent samples *t*-test on Week 8 absolute values.

All post-treatment values remain within established normal reference ranges.

### Adverse events

No untoward adverse events were reported in this investigation.

## Discussion

**Fig. 3:**
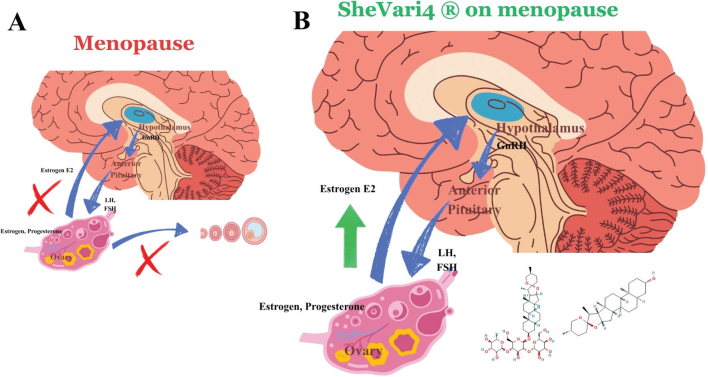
Generalized mechanism of endocrine dysfunction during menopause and subsequent restoration by SheVari4^®^ (a) During menopause, normal physiological estrogen signaling which operates via the hypothalamic-pituitary axis is impaired due to lack of estrogen production by the follicles of ageing ovaries. This leads to a significant drop in the levels of key reproductive hormones and subsequent inhibition of menstrual cycle (b) Key steroidal saponins such as Shatavarin IV present in SheVari4^®^ act as phytoestrogens to reinitiate the estrogen signalling leading to partial restoration of the endocrine balance and overall rejuvenation of menopausal health.

The effect of SheVari4^®^ was investigated in a population of women in a group of pre-, peri and postmenopausal women with normal BMI and cardiovascular parameters, which ensured that there were risk factors related to venous thrombosis and cardiovascular complications (35). SheVari4^®^ achieved statistically significant reductions in the MENQOL scores across all four domains with a substantially high standardized mean difference or Cohen’s D value (36). Secondary endpoint determination also suggested that SheVari4^®^ achieved its desired effect of raising the level of estradiol E2, the most important estrogen during reproductive years (37). Alongside, it also achieved a reduction in FSH levels. During normal reproductive cycle, ovulation is mediated by a strict estrogen mediated regulation through an interplay of negative and positive feedback loop (38). In the negative feedback loop, estrogen from the ovaries exerts inhibitory action over both the hypothalamus and the anterior pituitary during the early and middle follicular phase and the luteal phase to arrest the secretion of Gonadotrophin Releasing Hormone (GnRH) (from hypothalamus) as well as Luteinizing Hormone (LH) and FSH from the anterior pituitary (39). This ensures prevention of 1) release of premature follicles and 2) premature FSH surge and also leads to reduced estradiol, the major circulating estrogen during reproductive phase (40) and progesterone, released from the corpus luteum of the ovary. On the contrary, during the late follicular maturation phase, which falls typically within day 12 to 14 of each menstrual cycle, a high sustained release of estradiol is needed to increase the level of LH and subsequent burst of the follicles causing release of eggs (ovulation) (41). During perimenopause, as a consequence of aging of the ovaries, the follicles fail to produce the requisite estrogen to maintain this cycle and subsequently, the regulation of the Hypothalamic-Pituitary-Gonadal (HPG) axis is gradually worn out leading to irregular menstrual cycles which eventually stops completely marking the transition from perimenopause to menopause (42). Simultaneous restoration of estradiol E2 with a slight reduction of FSH as a result of regular consumption of SheVari4^®^ is suggestive of the fact that Shatavarin IV present in the formulation acted as a phytoestrogen to partially restore the regulation of the HPG axis and reverse the psychophysiological damage inflicted by the deficit of estrogen levels (Fig. 3).

In addition to achieving noteworthy restoration in the levels of the reproductive hormone, SheVari4^®.^ also resulted in a substantial decrease in cortisol levels. A major factor accounting for unsuccessful pregnancies, especially in recent times, has been rising levels of stress, both in home front as well as in workplace amongst couples (43). Cortisol, a steroid hormone secreted from the adrenal cortex, can jeopardize female reproductive health by altering endocrine balance leading to irregular menstrual cycles, impaired ovulation and subsequent infertility (44). It has been demonstrated from our previous studies that Shatavarin IV, a steroidal saponin, potentially targets the Receptor Tyrosine kinases mediating a plethora of signalling pathways involved in augmentation of neuronal health (30). Additional preliminary studies from Gene Ontology have predicted that a potential target of Shatavarin IV action is CYP17A1, a cytochrome P450 family enzyme with integral role in steroid hormone biosynthesis (45). The corresponding disease involvement database has revealed Cushing Syndrome, a hormonal disorder caused by prolonged exposure to high levels of cortisone (46). RTK mediated signalling pathways can directly influence the activity of hypothalamic neurons that release corticotropin-releasing hormone (CRH), a key regulator of the Hypothalamic Pituitary Adrenal axis (47, 48). CRH stimulates the release of adrenocorticotropic hormone (ACTH) from the pituitary gland, which in turn triggers the release of glucocorticoids (like cortisol) from the adrenal glands (49).Therefore, it is apparent that a strict control on regulation of cortisol levels is integral for successful pregnancies as well as for avoiding miscarriages. A gradual decline in the level of Anti Mullerian Hormone (AMH) is also an authentic indicator of a shrinking ovarian reserve leading to menopause (50). Administration of SheVari4^®^ led to a small but significant increase in AMH thus further justifying its intended potential. In harmony with the elevated levels of estrogen and progesterone, the level of Sex Hormone Binding Globulin (SHBG), a serum carrier of these hormones (51) was also increased in the SheVari4^®^ group. Together, the results suggested that SheVari4^®,^ with its enriched content of Shatavarin IV, achieved modest improvements in restoration of the endocrine balance in menopausal disorders.

The working dosage of 100 mg per day over a period of 8 consecutive weeks was found to be safe, as assessed by oral toxicity analyses as well as through assessment of liver and kidney function tests and evaluation of other clinical parameters. A decrease in levels of CK indicated that the formulation was effective in increasing muscle strength and endurance (52). Parallelly, reductions in the levels of SGPT, SGOT and ALP levels (within the normal range) were achieved, which indicated that the formulation demonstrated hepatoprotective activity; all liver enzyme values remained well within normal reference ranges throughout the treatment period (53). BUN level is an effective indicator of kidney health and its increase is often associated with a compromised metabolic status of the body (54). As a result of consumption of SheVari4^®^, BUN levels were also brought down, which reinstated its holistic wellness potential. Similar formulations of *A. racemosus* root extract had been previously reported to have alleviated menopausal discomforts successfully. However, in all of these cases, the net Shatavarin IV content available on methanolic extraction varied considerably from each other and did not necessarily correlated with the amount available for bioabsorption. Gudise et al. reported a formulation named Aspurūs™ with a Shatavarin IV content of 5% requiring a dosage of administration of 250 mg/day (55). A similar study carried out by Pingali et al. reported the use of another standardized root extract of Shatavari at a dosage of 250 mg/day alone or in conjunction with Ashwagandha root extract with a total saponin content of greater than 30%, on improvement of menopausal QOL (56). However, the content of Shatavarin IV, the most significant bioactive molecule of Shatavari, was not reported. Another study by O’Leary et al. reported the increase in handgrip strength in post-menopausal women after administration of Shatavari root extract at a dosage of 500 mg/day for a period of 6 weeks (57) but here also, the content of Shatavarin IV was undisclosed. A very recent study conducted on perimenopausal women reported improvement in QOL after administration of Shatavari root extract containing more than 10% total Shatavarins at a dosage of 300 mg/day over a period of 8 weeks (58). The formulation made in our laboratory with a proprietary technology contained 7% of Shatavarin IV on dissolution in methanol and the effective administered dosage was kept as low as 100 mg/day, thus safely avoiding any immediate or chronic toxicity issue.

## Conclusion

Although the phytotherapeutic potential of *A. racemosus*, especially with regard to revitalisation of menopausal health, has been reported earlier by several studies, the present formulation of SheVari4^®^ is one of the very few such studies with a significantly high concentration of Shatavarin IV, which is not only safe for administration but also achieves a holistic improvement in liver and kidney functions. Studied carried out in our laboratory had explored the mechanistic insights of Shatavarin IV integral to the health rejuvenating potential of this significant medicinal herb. The potential of Shatavari as a superior alternative to traditional allopathic and OTC drugs for treating menopausal symptoms can only be successfully disseminated once its full spectrum of cellular points of intervention has been deciphered.
